# Community-directed malaria freedom on Aneityum Island, Vanuatu, 1991-2014

**DOI:** 10.1186/1475-2875-13-S1-P50

**Published:** 2014-09-22

**Authors:** Akira Kaneko, Chim Chan, George Taleo, Noriko Watanabe, Morris Kalkoa, Sam Iamar, Luis F Chaves, Klara Junker, Jackie Cook, Rie Isozumi, Masatsugu Kimura, Chris Drakeley

**Affiliations:** 1 Karolinska Institutet, Stockholm, Sweden; 2 Osaka City University, Osaka, Japan; 3 Nagasaki University Institute of Tropical Medicine, Nagasaki, Japan; 4 Ministry of Health, Port Vila, Vanuatu; 5 London School of Hygiene and Tropical Medicine, London, UK

## Background

Weekly mass drug administration (MDA) of chloroquine, pyrimethamine/sulfadoxine, and primaquine was carried out on the entire population of 718 inhabitants of Aneityum Island for nine weeks in September - November 1991, before the onset of the rainy season. Simultaneously, insecticide-treated bednets (ITNs) were distributed to the entire population. Microscopy showed the immediate disappearance of *Plasmodium falciparum*, whereas *P. vivax* disappeared from 1996 onwards [Kaneko 2000] until new cases were reported in January 2002. In July 2002, *P. vivax* infections were detected by microscopy in 22 of 759 individuals: 20/298 born after 1991, 2/126 born between 1991-82, and 0/335 born before 1982. Subsequent PCR increased the total to 77 (36, 21, and 20 in respective age groups). The age distribution was similar to those before elimination and on other islands. In November, a similar age pattern was found but with fewer (39) infections [Kaneko 2014].

## Materials and methods

In December 2002, a second MDA of weekly chloroquine for four weeks and daily primaquine for 14 days was carried out as a containment measure against the *P. vivax* resurgence in the cohort born after 1982, in concert with re-strengthening of the community-based provision of ITNs. After that we monitored malaria prevalence on Aneityum by conducting yearly island-wide mass blood surveys.

## Results

PCR detected no positive cases in December 2002 (n = 436, only those born after 1982), immediately after the second MDA, but 26 in 2003 (730), 20 in 2004 (732), 34 in 2005 (836), and 15 in 2007 (719). No cases were detected in 2010 (950) and 2013 (1093). The age distribution of the 2003-2007 positive cases was different from those before the second MDA and on other islands, i.e. a substantially lower prevalence was observed in the cohort born after 2002 (0.8%) than those between 2002 and 1992 (3.7%), between 1991 and 1982 (5.3%), and before 1982 (2.1%), suggesting that most of these submicroscopic infections represented relapses from hypnozoites. Sero-epidemiological monitoring suggested that the persistence of antibodies against *P. vivax* may partially explain the lower parasite prevalence in the oldest age group. On Aneityum, indigenous *P. falciparum* transmission has never re-established after the first MDA in 1991, despite surveillance by community microscopists that showed continuous parasite importation from other islands.

## Conclusions

A high degree of community engagement to prevent resurgence [Kaneko 2010], in addition to high ITN coverage (1.05 net/person in 2014) and usage (95% in 2014), sustains malaria freedom on this island.

